# Denervated mouse CA1 pyramidal neurons express homeostatic synaptic plasticity following entorhinal cortex lesion

**DOI:** 10.3389/fnmol.2023.1148219

**Published:** 2023-04-12

**Authors:** Maximilian Lenz, Amelie Eichler, Pia Kruse, Phyllis Stöhr, Dimitrios Kleidonas, Christos Galanis, Han Lu, Andreas Vlachos

**Affiliations:** ^1^Department of Neuroanatomy, Institute of Anatomy and Cell Biology, Faculty of Medicine, University of Freiburg, Freiburg, Germany; ^2^Center BrainLinks-BrainTools, University of Freiburg, Freiburg, Germany; ^3^Center for Basics in Neuromodulation (NeuroModulBasics), Faculty of Medicine, University of Freiburg, Freiburg, Germany

**Keywords:** entorhinal cortex lesion, Schaffer collateral lesion, transcriptome analysis, microglia, RNA oxidation, synaptic plasticity

## Abstract

Structural, functional, and molecular reorganization of denervated neural networks is often observed in neurological conditions. The loss of input is accompanied by homeostatic synaptic adaptations, which can affect the reorganization process. A major challenge of denervation-induced homeostatic plasticity operating in complex neural networks is the specialization of neuronal inputs. It remains unclear whether neurons respond similarly to the loss of distinct inputs. Here, we used *in vitro* entorhinal cortex lesion (ECL) and Schaffer collateral lesion (SCL) in mouse organotypic entorhino-hippocampal tissue cultures to study denervation-induced plasticity of CA1 pyramidal neurons. We observed microglia accumulation, presynaptic bouton degeneration, and a reduction in dendritic spine numbers in the denervated layers 3 days after SCL and ECL. Transcriptome analysis of the CA1 region revealed complex changes in differential gene expression following SCL and ECL compared to non-lesioned controls with a specific enrichment of differentially expressed synapse-related genes observed after ECL. Consistent with this finding, denervation-induced homeostatic plasticity of excitatory synapses was observed 3 days after ECL but not after SCL. Chemogenetic silencing of the EC but not CA3 confirmed the pathway-specific induction of homeostatic synaptic plasticity in CA1. Additionally, increased RNA oxidation was observed after SCL and ECL. These results reveal important commonalities and differences between distinct pathway lesions and demonstrate a pathway-specific induction of denervation-induced homeostatic synaptic plasticity.

## Introduction

Neurons receive various short- and long-range inputs that are crucial for signal processing and normal brain functioning (Forster et al., 2006; Guy and Staiger, 2017; Shepherd and Rowe, 2017). Pathological conditions associated with neuronal damage and cell death, such as multiple sclerosis, stroke, or traumatic brain injury, lead to the denervation of intact brain regions. This poses consequences for proper network function (Navarro et al., 2007; O'Reilly et al., 2014; Henry et al., 2020; Sato et al., 2021; Beck et al., 2022), with adaptive processes triggered to compensate for the loss of input (Keck et al., 2008; Vlachos et al., 2013a; Becker et al., 2015; Sammons and Keck, 2015; Lenz et al., 2019). A hallmark of denervation-induced neuronal adaptations is the induction of homeostatic synaptic plasticity. Based on negative feedback mechanisms, homeostatic synaptic plasticity adjusts synaptic weights to maintain neuronal activity within a dynamic range (Davis and Bezprozvanny, 2001; Marder and Prinz, 2003; Pozo and Goda, 2010; Turrigiano, 2012; Keck et al., 2013; Bissen et al., 2021; Cao et al., 2022). Various functional and mechanical lesion models have been established to identify the cellular and molecular mechanisms of lesion-induced homeostatic synaptic plasticity (e.g., Nataraj et al., 2010; Vlachos et al., 2013a; Barnes et al., 2015; Lenz et al., 2019; Wen and Turrigiano, 2021; Kruse et al., 2022). However, a major challenge of denervation-induced homeostatic plasticity operating in complex networks is the structural, functional, and molecular specialization of neuronal input pathways, which leads to synaptic diversity (O'Rourke et al., 2012; c.f. Lee and Kirkwood, 2019; Grant and Fransen, 2020). Therefore, it is unclear whether neurons respond differently to the loss of distinct inputs under pathological conditions.

Previous research revealed that *in vitro* entorhinal cortex lesion (ECL) of mouse organotypic entorhino-hippocampal slice cultures induced homeostatic synaptic plasticity of excitatory synapses on dentate granule cells (Vlachos et al., 2012; Lenz et al., 2019). These functional changes depended on cytokine signaling (Becker et al., 2015) and involved the structural remodeling of denervated dendrites (Vlachos et al., 2012, 2013b; Schuldt et al., 2016; Willems et al., 2016; Bissen et al., 2021), similar to what is observed after *in vivo* ECL (Vuksic et al., 2011). Moreover, the induction of homeostatic synaptic plasticity was demonstrated to affect the post-lesional recovery of dendritic spine numbers (Vlachos et al., 2013b). Recently, we showed that microglia delineate the denervated dentate gyrus after ECL (Kleidonas and Vlachos, 2021), and that ECL triggers homeostatic synaptic changes in CA3 pyramidal neurons (Kruse et al., 2022).

In this study we used ECL and Schaffer collateral lesion (SCL; c.f. McKinney et al., 1999) of mouse entorhino-hippocampal slice cultures to test for pathway-specific structural, functional, and molecular adaptations of CA1 pyramidal neurons. Both lesions led to microglia accumulation, degeneration of presynaptic boutons at asymmetric synapses, and dendritic spine loss in the denervated CA1 layers. Transcriptomic analysis identified commonalities and differences between SCL and ECL, thereby implying the existence of a pathway-specific induction of denervation-induced synaptic changes. Indeed, functional and mechanical ECL induced homeostatic plasticity of CA1 excitatory synapses. Furthermore, a significant increase in oxidative RNA modifications was observed after SCL and ECL.

## Materials and methods

### Ethics statement

Mice were maintained in a 12 h light/dark cycle with food and water available *ad libitum*. Every effort was made to minimize distress and pain of animals. All experimental procedures were performed according to the German animal welfare legislation and approved by the animal welfare committee and/or the animal welfare officer at the University of Freiburg, Faculty of Medicine (X-17/07K, X-18/02C, X-21/01B).

### Preparation of organotypic tissue cultures

Entorhino-hippocampal tissue cultures were prepared at postnatal day 4–5 from C57BL/6J and Thy1-eGFP (Feng et al., 2000) animals of either sex as previously described (Del Turco and Deller, 2007). Cultivation medium contained 50% (v/v) MEM, 25% (v/v) basal medium eagle, 25% (v/v) heat-inactivated normal horse serum, 25 mM HEPES buffer solution, 0.15% (w/v) bicarbonate, 0.65% (w/v) glucose, 0.1 mg ml^−1^ streptomycin, 100 U ml^−1^ penicillin, and 2 mM glutamax. The pH was adjusted to 7.3 and the medium was replaced three times per week. All tissue cultures were allowed to mature for at least 18 days in a humidified atmosphere with 5% CO_2_ at 35°C, since at this stage a steady-state in structural and functional properties of the organotypic tissue cultures is reached (Hailer et al., 1996; Vlachos et al., 2012, 2013c; Humpel, 2015).

### Mechanical pathway lesion

**Figure 1:**
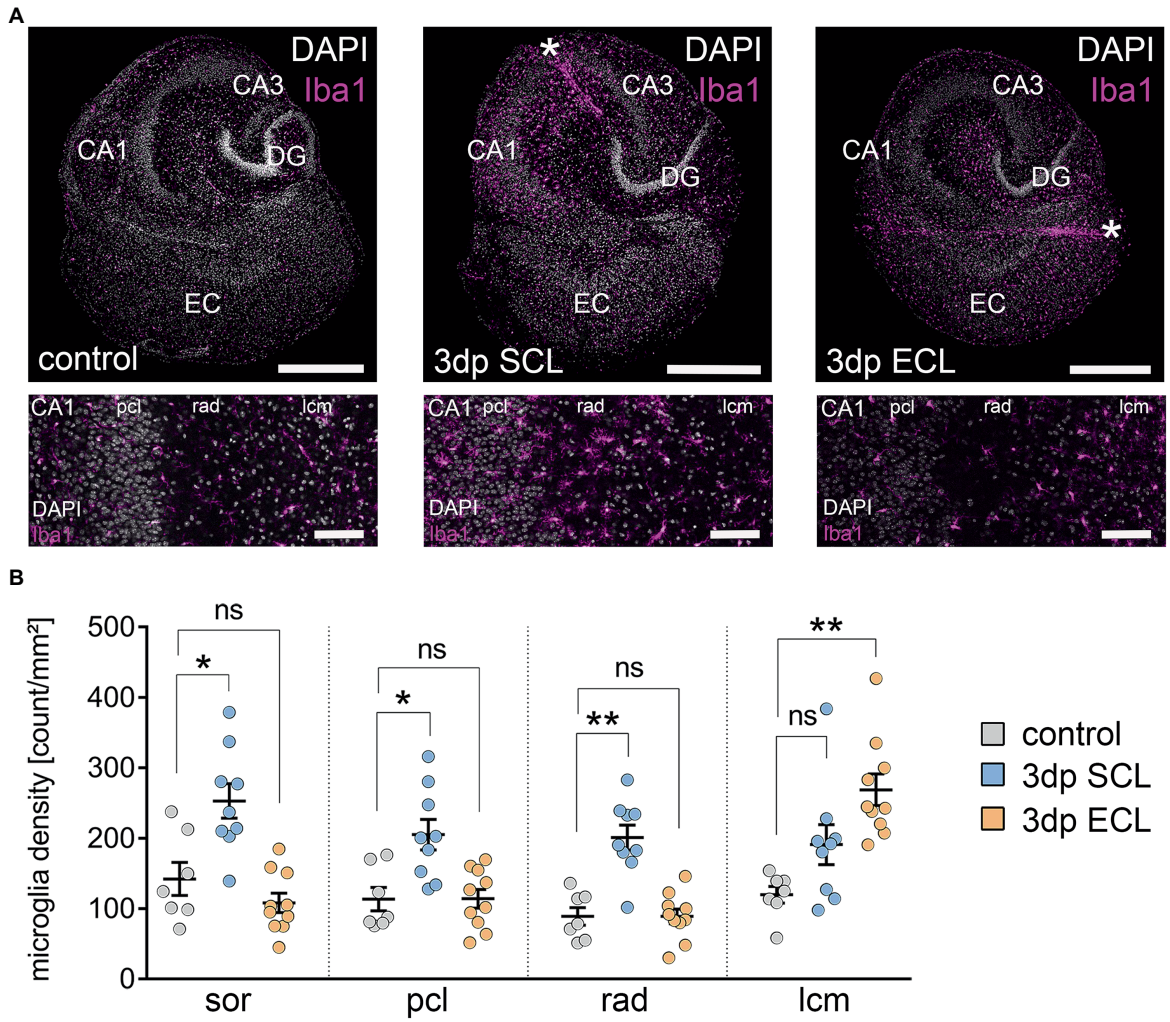
SCL and ECL both lead to layer-specific microglia accumulation in the CA1 region. **(A)** Representative examples of entorhino-hippocampal tissue cultures in non-lesioned controls (left panel) and 3 days after performing Schaffer collateral lesion (SCL, middle panel) or entorhinal cortex lesion (ECL, right panel) stained with DAPI nuclear stain and for the microglia marker Iba1 (EC, entorhinal cortex; DG, dentate gyrus; 3dp, 3 days post lesion; *, lesion). Higher magnifications (bottom row) illustrate the laminar accumulation of microglia in the CA1 region upon lesion (pcl, pyramidal cell layer; rad, str. radiatum; lcm, str. lacunosum-moleculare). Scale bar, 600 μm (upper panels) and 100 μm (bottom panels). **(B)** Microglia density was significantly increased in the str. oriens (sor), str. pyramidale (pcl) and str. radiatum (rad) following SCL while it remained unchanged in these layers after ECL. However, in the str. lacunosum-moleculare (lcm) microglia density was specifically increased following ECL but not SCL (n_control_ = 7 cultures, n_SCL_ = 9 cultures, n_ECL_ = 10 cultures, Kruskal-Wallis test followed by Dunn’s posthoc correction). Individual data points are indicated by colored dots. Values represent mean ± s.e.m. (*p < 0.05, **p < 0.01, ns - non significant difference).

Mechanical pathway transection was performed with a sterile scalpel in mature tissue cultures (≥ 18 days *in vitro*). To apply an entorhinal cortex lesion (ECL), the perforant path was transected from the rhinal to the hippocampal fissure (Figure 1A). Schaffer collateral lesion (SCL) was applied between the CA3 and the CA1 region of the hippocampus, without affecting the perforant path projections to CA3 (Figure 1A). Except for the lesion-induced partial denervation of CA1 pyramidal neurons, cytoarchitecture of both the hippocampus and the entorhinal cortex remained unchanged.

### Chemogenetic pathway silencing

pAAV-hSyn-hM4D (Gi)-mCherry was a gift from Bryan Roth (Addgene viral prep #-50,475-AAV2; http://n2t.net/addgene:50475; RRID: Addgene_50,475; 7 × 10^12^ vg/ml, 1:4 diluted in PBS). AAV was injected into the CA3 region or the entorhinal cortex at 3–5 days *in vitro* using borosilicate glass pipettes (c.f. Lenz et al., 2019, 2022). Cultures were returned to the incubator immediately after injection and allowed to mature for at least 18 days in a humidified atmosphere with 5% CO_2_ at 35°C. Silencing was achieved by clozapine *N*-oxide treatment (CNO; 100 μM, 2 days; Tocris, #4936). Vehicle-only treatment (0.1% (v/v) DMSO) served as control in these experiments.

### Immunohistochemistry

Cultures were fixed in a solution of 4% (w/v) paraformaldehyde (PFA) in phosphate-buffered saline (PBS, 0.1 M, pH 7.4) and 4% (w/v) sucrose for 1 h. Fixed cultures were incubated for 1 h with 10% (v/v) normal goat serum (NGS) in 0.5% (v/v) Triton X-100-containing PBS to block non-specific staining. Whole tissue cultures were incubated with rabbit anti-Iba1 (1:1000; Fujifilm Wako, #019–19741) or mouse anti-DNA/RNA oxidative damage (8-hydroxyguanosine (oh8G); 1:1000; QED Bioscience, #12501), in PBS containing 10% (v/v) normal goat serum (NGS) and 0.1% (v/v) Triton X-100 at 4°C overnight. Cultures were washed and incubated for 3 h with appropriate secondary antibodies (1:1000, in PBS with 10% NGS or NHS, 0.1% Triton X-100; Invitrogen). DAPI nuclear stain (1:5000 in PBS for 10 min; Thermo Scientific, #62248) was used to visualize cytoarchitecture. Sections were washed, transferred onto glass slides and mounted for visualization with anti-fading mounting medium (DAKO Fluoromount).

Confocal images in immunostainings were acquired using a Leica SP8 confocal microscope equipped with a 20x (NA 0.75, Leica) or 40x (NA 1.3, Leica) objective lens. Detector gain and amplifier were initially set to obtain pixel intensities within a linear range.

### Posthoc-staining

Cultures were fixed in a solution of 4% (w/v) paraformaldehyde (PFA) in phosphate-buffered saline (PBS, 0.1 M, pH 7.4) and 4% (w/v) sucrose for 1 h. Fixed cultures were incubated for 1 h with 10% (v/v) normal goat serum (NGS) in 0.5% (v/v) Triton X-100-containing PBS. Biocytin filled cells were counterstained with Alexa 647-conjugated streptavidin (1:1000 in PBS with 10% NGS, 0.1% Triton X-100; Invitrogen, #S-32357) for 4 h and DAPI staining was used to visualize cytoarchitecture (1:5000 in PBS for 10 min; Thermo Scientific, #62248). Slices were washed, transferred, and mounted onto glass slides for visualization with anti-fading mounting medium (DAKO Fluoromount). Confocal images were acquired using a Leica SP8 confocal microscope equipped with a 20x objective lens (NA 0.75, Leica).

### Transmission electron microscopy

Tissue cultures were fixed in 4% paraformaldehyde (w/v) and 2% glutaraldehyde (w/v) in 0.1 M phosphate buffer (PB) overnight and washed for 1 h in 0.1 M PB. After fixation, tissue cultures were incubated with 1% osmium tetroxide for 20 min in 5% (w/v) sucrose containing 0.1 M PB. The slices were washed 5 times for 10 min in 0.1 M PB and washed in graded ethanol [10 min in 10% (v/v) and 10 min in 20% (v/v)]. The slices were then incubated with uranyl acetate (1% (w/v) in 70% (v/v) ethanol) overnight and subsequently dehydrated in graded ethanol 80% (v/v), 90% (v/v) and 98% (v/v) for 10 min. Finally, slices were incubated with 100% (v/v) ethanol two times for 15 min followed by two 15 min washes with propylene oxide. The slices were then transferred for 30 min in a 1:1 mixture of propylene oxide with durcupan and then for 1 h in durcupan. The durcupan was exchanged for fresh durcupan and the slices were transferred to 4°C overnight. The slices were then embedded between liquid release-coated slides and coverslips. Cultures were re-embedded in blocks and ultrathin sections were collected on copper grids. Electron microscopy was performed with a LEO 906E microscope (Zeiss) at 3596x magnification. Acquired images were saved as TIF-files and analyzed using the ImageSP Viewer software.^1^ Asymmetric spine synapses were identified and manually quantified by an investigator blind to experimental conditions and hypotheses.

### Whole-cell patch-clamp recordings of excitatory neurotransmission

Whole-cell voltage-clamp recordings from CA1 pyramidal neurons of slice cultures were carried out at 35°C (2–5 neurons per culture). The bath solution contained 126 mM NaCl, 2.5 mM KCl, 26 mM NaHCO_3_, 1.25 mM NaH_2_PO_4_, 2 mM CaCl_2_, 2 mM MgCl_2_, and 10 mM glucose. For EPSC recordings patch pipettes contained 126 mM K-gluconate, 4 mM KCl, 4 mM Mg-ATP, 0.3 mM Na_2_-GTP, 10 mM phosphocreatine, 10 mM HEPES, and 0.3% (w/v) biocytin (pH = 7.25 with KOH, 290 mOsm with sucrose) having a tip resistance of 4–6 MΩ. Cells were visually identified using an LN-Scope (Luigs and Neumann, Ratingen, Germany) equipped with infrared dot-contrast and a 40x water-immersion objective (NA 0.8, Olympus). Electrophysiological signals were amplified using a Multiclamp 700B amplifier, digitized with a Digidata 1550B digitizer, and visualized with the pClamp 11 software package. Spontaneous excitatory postsynaptic currents (sEPSCs) of CA1 pyramidal neurons were recorded in voltage-clamp mode at a holding potential of-60 mV. In chemogenetic silencing experiments, miniature EPSCs (mEPSCs) were recorded to avoid an increase of synaptic activity in silenced pathways. For mEPSC recordings, D-APV (10 μM; Abcam, #ab120003), tetrodotoxin (TTX, 0.5 μM; Biotrend, #18660–81-6) and bicuculline-methiodide (10 μM; Abcam, #ab120108) were added to the external solution and the holding potential was set to −70 mV. Series resistance was monitored before and after each recording and recordings were discarded if the series resistance reached ≥30 MΩ.

### Regional mRNA library preparation and transcriptome analysis

RNA library preparations for transcriptome analysis were performed using the NEBNext® Single Cell/Low Input RNA Library Prep Kit for Illumina® (New England Biolabs, #E6420) according to the manufacturer’s instructions. Briefly, isolation of the CA1 region from individual tissue cultures was performed from non-lesioned control, SCL and ECL cultures 3days after the lesion using a scalpel without collecting the scar tissue at the lesion site. The tissue of one single isolated CA1 region was transferred to 7.5 μl lysis buffer (supplemented with murine RNase inhibitor) and homogenized using a pestill. Samples were centrifuged for 30 s at 10,000 *g* and 5 μl of supernatant were collected from individual samples and further processed. After cDNA synthesis, cDNA amplification was performed according to the manufacturer’s protocol. The cDNA yield was subsequently analyzed by a High Sensitivity DNA assay on a Bioanalyzer instrument (Agilent). The amount of cDNA was adjusted to 10 ng for further downstream applications. After fragmentation and adaptor ligation, dual index primers (New England Biolabs, #E7600S) were ligated in a library amplification step using 10 PCR cycles. Libraries were finally cleaned up with 0.8X SPRI beads following a standard bead purification protocol. Library purity and size distribution were assessed with a High Sensitivity DNA assay on a Bioanalyzer instrument (Agilent). We quantified the libraries using the NEBNext Library Quant Kit for Illumina (New England Biolabs, #E7630) based on the mean insert size provided by the Bioanalyzer. A 10 nM sequencing pool (120 μl in Tris-HCl, pH 8.5) was generated for sequencing on the NovaSeq6000 sequencing platform (Illumina; service provided by CeGaT GmbH, Tübingen, Germany). We performed a paired-end sequencing with 150 bp read length. Data analysis was performed at the Galaxy platform (usegalaxy.eu; Galaxy and Galaxy, 2022). All files contained more than 10 M high-quality reads (after mapping to the reference genome mm10) with a phred quality of at least 30 (>90% of total reads).

### Time lapse imaging of dendritic spines in CA1 pyramidal neurons

Time lapse imaging of dendritic spines was performed in Thy1-eGFP tissue cultures at a Zeiss LSM800 microscope equipped with a 10x (NA 0.3; Carl Zeiss) and a 60x objective (NA 1.0; Carl Zeiss). Filter membranes with 3–6 cultures were placed in a 35 mm Petri Dish containing pre-warmed and-oxygenated imaging solution consisting of 50% (v/v) MEM, 25% (v/v) BME, 50 mM HEPES buffer solution (25%, v/v), 0.65% (w/v) glucose, 0.15% (w/v) bicarbonate, 0.1 mg/ml streptomycin, 100 U/ml penicillin, 2 mM glutamax, and 0.1 mM trolox. The cultures were kept at 35°C during the imaging procedure. Baseline imaging was performed immediately before applying SCL or ECL, respectively. Equally handled non-lesioned cultures served as control cultures. Laser intensity and detector gain were initially set to keep the fluorescent signal in a dynamic range throughout the experiment and were kept constant during time series. For each dendritic layer in the CA1 region, a z-stack containing multiple dendritic segments was recorded before lesion (day 0) with Δz = 0.4 μm at ideal Nyquist rate and an optical zoom of 1. After imaging, lesions were applied and cultures were returned to the incubator. The imaging procedure was repeated 3 days after the lesion (day 3) following the same experimental protocol with the same imaging parameters. Confocal image stacks were stored as .czi files.

### Quantification and statistics

In this study, we used age-matched organotypic entorhino-hippocampal tissue cultures of either sex in a prospective study design to elucidate SCL-and ECL-induced plasticity in the CA1 region with non-lesioned cultures serving as controls. Electrophysiological data were analyzed using pClamp 10.7 (Axon Instruments) software. EPSC properties were analyzed using the automated template search tool for event detection (Lenz et al., 2021).

Microglial density was assessed by counting immunostained microglia manually in defined dendritic layers. The fluorescence signal of immunostained oxRNA was assessed in dendritic layers of the CA1 region in single-plane images. To avoid the detection of somatic signals, the regions of interest (ROIs) were placed in areas that did not contain any nuclei or somata. All analyses using immunohistochemistry were performed by investigators blind to experimental conditions.

Dendritic spine density was determined by the automated detection tool ‘Spine Density Counter’ (Mighty-Data-Inc/dendritic-spine-counter: Dendritic Spine Counter v1.4.1 (v1.4.1). Zenodo. DOI: 10.5281/zenodo.6712248) in the Fiji software environment. Z-stacked fluorescent images were projected at maximum intensity to create a 2D representation of individual dendritic segments. ImageJ plugin ‘Spine Density Counter’ was used to detect spines, count spine numbers, measure segment length and to subsequently calculate spine density. For one dendritic segment imaged at different time points, special attention was paid to ensure that the same starting and ending points at the respective segment were used; the same pixel resolution was applied in the algorithm for spine detection in all images. Posthoc visual inspection was applied to ensure the spine detection results were not strongly-biased. Both raw spine density and normalized spine density to baseline were used in the analysis.

Synaptic degeneration was analyzed in electron micrographs of the dendritic layers in the CA1 region. Membrane disintegration, swelling and the extensive loss of synaptic vesicles were considered as signs for degeneration. Analysis was performed manually by an investigator blind to experimental conditions.

RNA sequencing data were uploaded to the galaxy web platform (public server: usegalaxy.eu; Afgan et al., 2016, 2018; Jalili et al., 2020) and transcriptome analysis was performed using the Galaxy platform in accordance with the reference-based RNA-seq data analysis tutorial (Batut et al., 2021). Adapter sequences, low quality, and short reads were removed *via* the CUTADAPT tool (Galaxy version 3.5 + galaxy0). Reads were mapped using RNA STAR (Galaxy version 2.7.8a + galaxy0) with the mm10 full reference genome (Mus musculus). The evidence-based annotation of the mouse genome (GRCm38), version M25 (Ensembl 100) served as gene model (GENCODE). For an initial assessment of gene expression, unstranded FEATURECOUNT (Galaxy version 2.0.1 + galaxy2) analysis was performed from RNA STAR output. Only samples that contained >60% uniquely mapping reads (feature: “exon”) were considered for further analysis. Genes with a low number of mean reads (base mean < 150 counts) were excluded from further analysis. Read counts were further analyzed using DESeq2. The functional enrichment analysis was performed using g:Profiler (version e107_eg54_p17_bf42210) with g:SCS multiple testing correction method applying significance threshold of 0.05 (Raudvere et al., 2019). Gene sets with 50–500 terms were considered for illustration.

Data were statistically analyzed using GraphPad Prism 9 (GraphPad software, USA). For statistical comparison of two unpaired experimental groups, a Mann–Whitney test was applied. For statistical comparison of two paired experimental groups (time-lapse imaging data sets), we used the Wilcoxon matched-pair signed rank test in normalized and non-normalized data. For the evaluation of data sets with three experimental groups, a Kruskal-Wallis test followed by Dunn’s posthoc correction was applied. Amplitude/frequency plots were statistically assessed by the repeated measure (RM) two-way ANOVA test with Sidak’s (two groups) multiple comparisons test. mEPSC frequency values from individual cells were stacked in subcolumns and mEPSC amplitude bins defined tabular rows (COLUMN factor: treatment; ROW factor: amplitude bin). *p*-values <0.05 were considered statistically significant (*p < 0.05, **p < 0.01, ***p < 0.001). The n-numbers are provided in the figure legends. Results that did not yield significant differences were designated ‘ns’. Statistical differences from RM-two-way ANOVA test in XY-plots were indicated in the legend of the figure panels (*) when detected through multiple comparisons. In the text and figures, values represent the mean ± standard error of the mean (s.e.m.).

### Data and material availability

Source data with statistical evaluations for each figure are provided on the Dryad data repository.^2^ Raw data (.fastq-files) used for transcriptome analysis are available at the Gene Expression Omnibus; accession number GSE223096. Original data are available from the corresponding authors upon reasonable request.

### Digital illustrations

Confocal image stacks were stored as TIF-files. Figures were prepared using the ImageJ software package^3^ and Photoshop graphics software (Adobe, San Jose, CA, USA). Image brightness and contrast were adjusted.

## Results

### Microglia accumulation in the denervated layers after SCL and ECL

SCL and ECL were performed in three-week old mouse organotypic entorhino-hippocampal tissue cultures using a sterile scalpel (Figure 1). After 3 days, Iba1-positive microglia delineated the lesion sites and accumulated in the denervated CA1 layers (Figure 1A). SCL triggered a significant increase in microglia numbers in the stratum oriens (sor), stratum pyramidale (pcl) and stratum radiatum (rad). In contrast, microglia density increased significantly in stratum lacunosum-moleculare after ECL (lcm; Figure 1B; c.f. Kleidonas and Vlachos, 2021). We concluded that both SCL and ECL triggered microglia accumulation in the denervated regions.

### Degeneration of presynaptic boutons in the denervated layers after SCL and ECL

**Figure 2:**
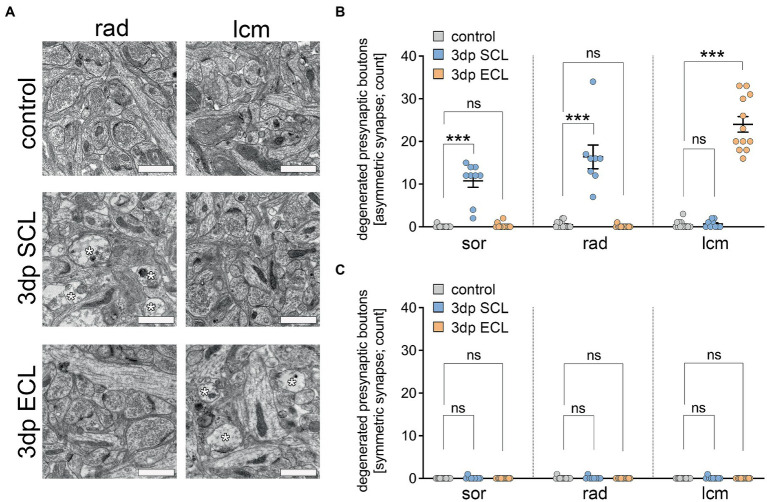
SCL and ECL lead to layer-specific degeneration of asymmetric synapses in the CA1 region. **(A)** Transmission electron microscopy was used to visualize a layer-specific degeneration of asymmetric synapses (membrane disintegration, swelling, loss of synaptic vesicles; white asterisks) in the CA1 region of entorhino-hippocampal tissue cultures. Scale bar, 1 μm. **(B)** Following SCL, a significant degeneration of presynaptic boutons of asymmetric synapses was evident in both str. oriens (sor) and str. radiatum (rad) but not in the str. lacunosum-moleculare (lcm). However, following ECL reversed effects were observed, e.g., a degeneration of asymmetric synapses in the lcm but not in either sor or rad (upper panel, n_control_ = 15 images, n_SCL_ = 9 images, n_ECL_ = 12 images from 3 cultures each, Kruskal-Wallis test followed by Dunn’s posthoc correction; in rad (SCL), one image was excluded from analysis due to preparation artifacts). **(C)** In presynaptic boutons of symmetric synapses no degeneration was detectable following either SCL or ECL (lower panel, n_control_ = 15 images, n_SCL_ = 9 images, n_ECL_ = 12 images from 3 cultures each, Kruskal-Wallis test followed by Dunn’s posthoc correction; in rad (SCL), one image was excluded from analysis due to preparation artifacts). Individual data points are indicated by colored dots. Values represent mean ± s.e.m. (***p < 0.001, ns - non significant difference).

Ultrastructural properties of CA1 synapses were assessed under control conditions and 3 days after SCL and ECL (Figure 2). Consistent with a lesion-induced loss of excitatory input, signs of presynaptic degeneration at asymmetric synapses (i.e., membrane disintegration, swelling and loss of synaptic vesicles) were observed in the denervated layers (Figure 2A). The number of degenerating presynaptic boutons in sor and rad significantly increased after SCL (Figure 2B). In contrast, presynaptic boutons degenerated in lcm after ECL, with sor and rad unaffected. These findings corroborated the *in vivo*-like fiber-and cytoarchitecture of entorhino-hippocampal tissue cultures (c.f. Lenz et al., 2022) and correlated well with the pathway-specific accumulation of microglia in the denervated layers (Figure 1). Notably, symmetric (inhibitory) synapses were unaffected 3 days after SCL and ECL (Figure 2C; c.f. Dinocourt et al., 2011; Lenz et al., 2019).

### Reduction in dendritic spine numbers in the denervated layers after SCL and ECL

**Figure 3:**
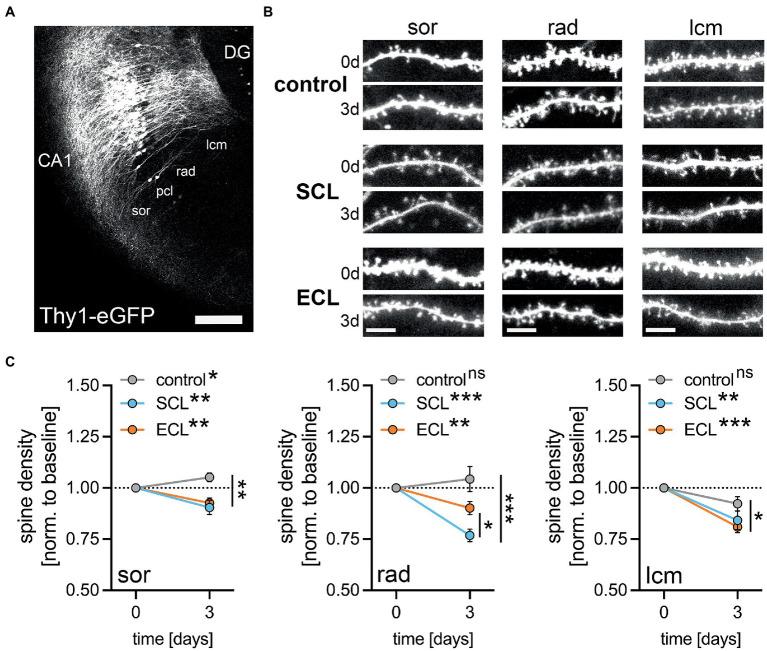
Lesion is accompanied by a loss of dendritic spines in CA1 pyramidal neurons. **(A)** CA1 region of an entorhino-hippocampal tissue culture from Thy1-eGFP mice (DG, dentate gyrus; sor, str. oriens; pcl, str. pyramidale; rad, str. radiatum; lcm, str. lacunosum-moleculare). Scale bar, 200 μm. **(B)** Layer-specific time-lapse images of CA1 dendrites at day 0 (0d) and day 3 (3d) following SCL or ECL, respectively. Scale bar, 5 μm. **(C)** Both SCL and ECL lead to a significant, yet layer-unspecific, decrease in spine density in hippocampal CA1 neurons. However, the magnitude of spine loss in rad showed significant differences between the lesions, where SCL caused a more prominent reduction than ECL (*p* = 0.02; sor: n_control_ = 37 dendritic segments, n_SCL_ = 24 dendritic segments, n_ECL_ = 34 dendritic segments, rad: n_control_ = 23 dendritic segments, n_SCL_ = 29 dendritic segments, n_ECL_ = 21 dendritic segments, lcm: n_control_ = 15 dendritic segments, n_SCL_ = 11 dendritic segments, n_ECL_ = 20 dendritic segments, Wilcoxon matched-pair signed rank test for time-dependent changes and Kruskal-Wallis test followed by Dunn’s posthoc correction for endpoint comparisons). Values represent mean ± s.e.m. (*p < 0.05, **p < 0.01, ***p < 0.001, ns - non significant difference).

We used time-lapse imaging of dendrites of CA1 pyramidal neurons in Thy1-eGFP cultures and assessed the denervation-induced changes in dendritic spine numbers (Figure 3A; c.f. Vlachos et al., 2012, 2013b; Bissen et al., 2021). Confocal image stacks were obtained from dendritic segments in sor, rad, and lcm in non-lesioned control cultures and shortly before SCL or ECL. Three days later, the same dendritic segments were re-identified, and changes in dendritic spine number were analyzed (Figure 3B). Spine numbers were stable under control conditions in rad and lcm, while a slight increase was observed in sor over time (Figure 3C). Dendritic spine numbers decreased after the lesions in all layers over time, irrespective of the lesioned pathway (Figure 3C). However, differences in the layer-specific endpoints of synaptic degeneration were observed. While SCL led to a significantly stronger decrease in spine density in rad compared to ECL, only ECL led to a significant loss of dendritic spines in lcm compared to the experimental endpoints in non-lesioned controls (Figure 3C). We concluded that SCL and ECL triggered similar events in different denervated layers, i.e., degeneration of excitatory boutons, microglia accumulation, and dendritic spine remodeling.

### Transcriptomic changes in CA1 after SCL and ECL

**Figure 4:**
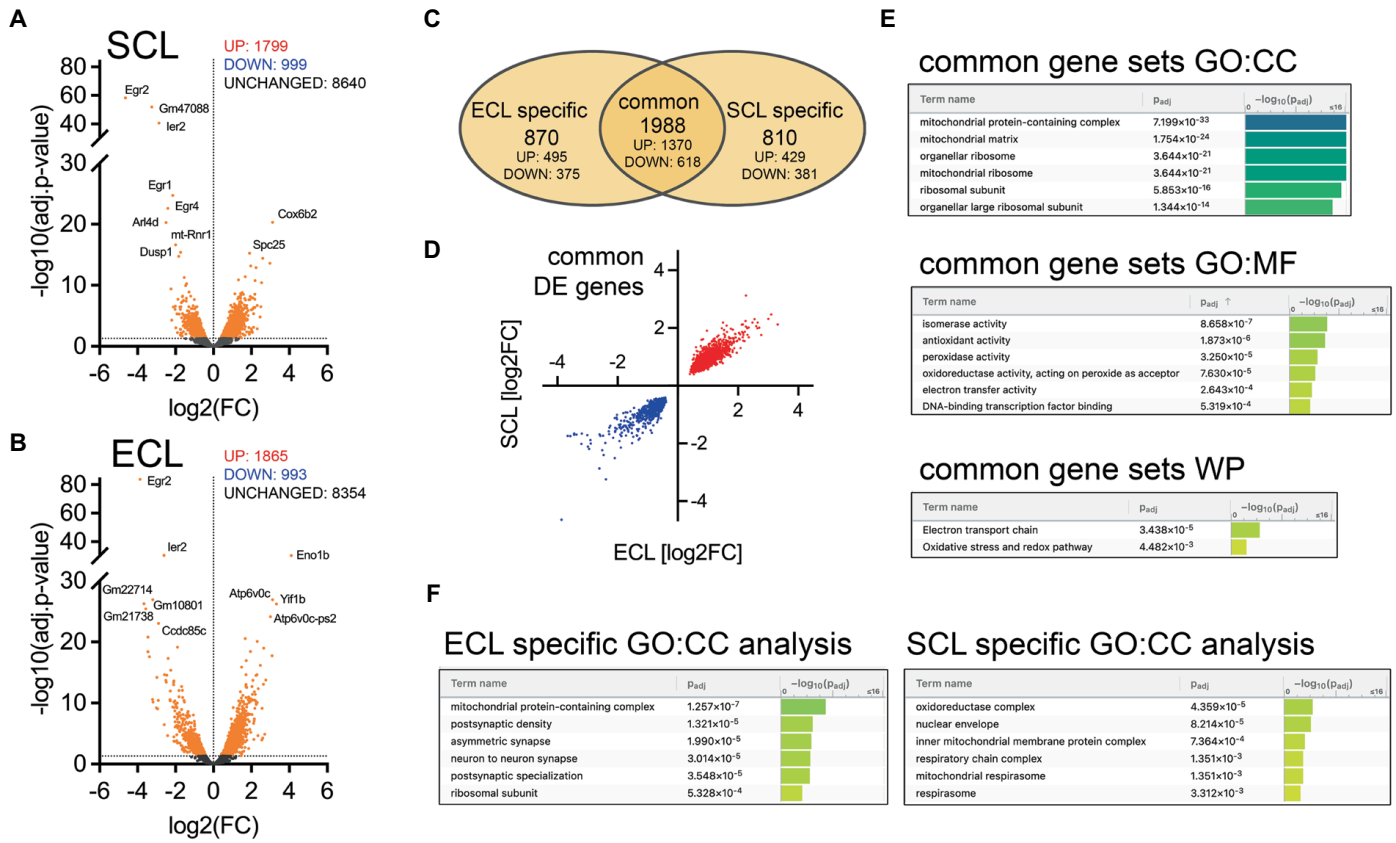
Lesion leads to pathway-specific transcriptomic changes in the hippocampal CA1 region. **(A)** Volcano plot illustrating differential gene expression in the CA1 region of non-lesioned cultures vs. 3 days following SCL (n_control_ = 5 cultures, n_SCL_ = 4 cultures). The significance threshold [*p*_adj._ < −log10(0.05)] is indicated by a dashed line. Significantly regulated genes are marked in orange and the top-10 regulated genes are labeled. **(B)** Volcano plot illustrating differential gene expression in the CA1 region of control cultures vs. 3 days following ECL (n_control_ = 5 cultures, n_ECL_ = 4 cultures). The significance threshold [*p*_adj._ < −log10(0.05)] is indicated by a dashed line. Significantly regulated genes are marked in orange and the top-10 regulated genes are labeled. **(C)** VENN-diagram to illustrate commonalities and differences in ECL-and SCL-induced differential gene expression. Although a substantial amount of commonly regulated genes could be identified, lesion-specificity became evident. **(D)** XY-plot of log2FC values from differentially expressed genes in both groups (‘common’). Notably, we exclusively found concordantly up- (red) and downregulated (blue) genes. **(E)** Functional enrichment analysis of common differentially expressed genes using the web-based g:Profiler platform [gene ontologies: CC - cellular compartment (TOP-6), MG - molecular function (TOP-6), and WP - wikipathways (all enrichments illustrated)]. **(F)** Functional enrichment analysis of lesion-specific differentially expressed genes. Notably, enrichment of differentially expressed synaptic gene sets was exclusively found after ECL.

To gain a better understanding of lesion-induced compensatory changes in the CA1 region at the molecular level, we conducted transcriptome analysis of the CA1 region to assess the commonalities and differences between transcriptomic profiles in non-lesioned controls and 3 days after SCL and ECL (Figure 4). Several differentially expressed genes were identified in the SCL and ECL groups (Figures 4A,B) compared to non-lesioned controls (further details provided in Supplementary Tables S1, S2). In total, a number of 1988 genes were commonly regulated after SCL and ECL, while 870 genes were specifically regulated after ECL and 810 genes were identified that changed after SCL only (Figure 4C; Supplementary Tables S3–S5). Notably, no discordantly regulated genes were identified among the commonly regulated genes 3 days after the lesions (Figure 4D). To identify the structural and functional targets of lesion-induced transcriptomic changes, we performed a gene set enrichment analysis (Figures 4E,F). This involved identifying relevant targets based on the number of differentially expressed genes in distinct gene ontology terms. The analysis of significantly regulated common gene sets showed that SCL and ECL induced distinct clusters related to mitochondrial function and oxidative stress (Figure 4E). Furthermore, the enrichment analysis of lesion-specific changes in gene expression revealed substantial differences (Figure 4F): SCL caused specific changes related to oxidative modifications, whereas ECL caused specific enrichment in synapse-related terms. These observations suggest lesion-specific effects on synaptic transmission. However, when we considered all differentially expressed genes, we found that synapse-related genes were enriched after both SCL and ECL (Supplementary Figure S1). We concluded that SCL and ECL induced complex transcriptomic changes with several concordantly regulated genes (including synapse-related genes). Additionally, lesion-specific differences were observed, suggesting that pathway-specific transcriptomic changes occured after SCL and ECL that reflect distinct functional states of synapses and networks. We followed up on these findings by testing for the induction of homeostatic synaptic plasticity and assessing denervation-induced oxidative changes, i.e., RNA oxidation, 3 days after SCL and ECL.

### Denervation-induced homeostatic strengthening of excitatory synapses after ECL

**Figure 5:**
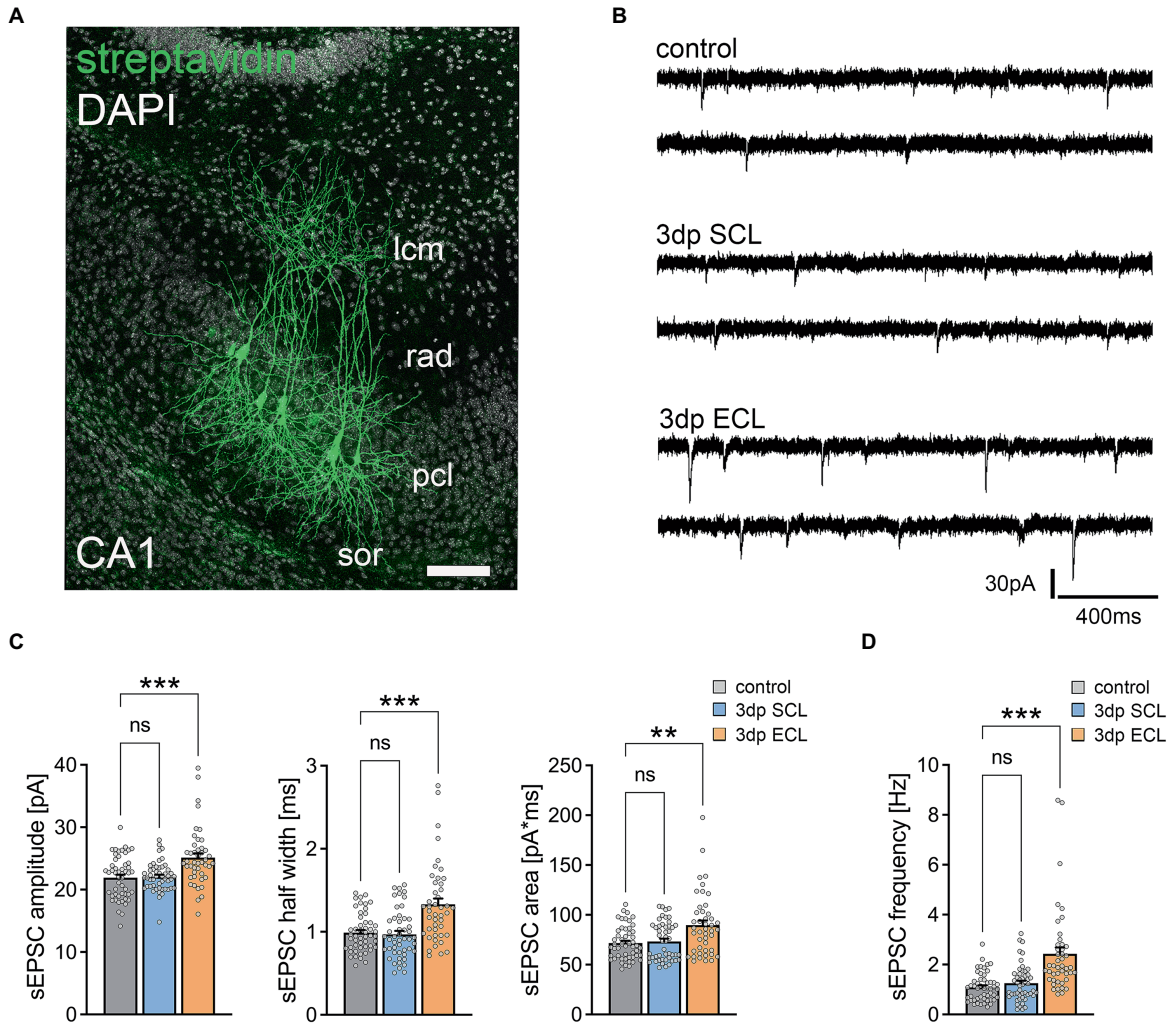
ECL but not SCL induces homeostatic synaptic plasticity in CA1 pyramidal neurons. **(A)** Posthoc visualization of CA1 pyramidal neurons after electrophysiological assessment (sor, str. oriens; pcl, str. pyramidale; rad, str. radiatum; lcm, str. lacunosum-moleculare). Scale bar, 100 μm. **(B)** Sample traces of spontaneous excitatory postsynaptic currents (sEPSC) recorded in hippocampal CA1 cells in non-lesioned control cultures as well as 3 days after either SCL or ECL (3dp, 3 days post lesion). **(C,D)** While sEPSC amplitude, half width and area as well as sEPSC frequency **(D)** were significantly increased in CA1 pyramidal neurons 3 days after ECL, no such effects can be observed following SCL (n_control_ = 51 cells in 14 cultures, n_SCL_ = 49 cells in 13 cultures, n_ECL_ = 45 cells in 12 cultures, Kruskal-Wallis test followed by Dunn’s posthoc correction). Individual data points are indicated by colored dots. Values represent mean ± s.e.m. (**p < 0.01, ***p < 0.001, ns - non significant difference).

The ECL-specific enrichment of synapse-related genes indicated potential differences in denervation-induced homeostatic synaptic plasticity following SCL and ECL (Figure 5). Therefore, we recorded AMPA-receptor-mediated spontaneous excitatory postsynaptic currents (sEPSCs) from individual CA1 pyramidal neurons in non-lesioned control cultures and 3 days after SCL and ECL (Figures 5A,B). While no significant changes in excitatory neurotransmission were observed in the SCL group, significant increases in mean sEPSC amplitude, half width, area, and frequency were observed in the ECL group (Figures 5C,D). We concluded that ECL triggered denervation-induced homeostatic synaptic plasticity consistent with a pathway-specific induction of homeostatic synaptic responses.

### Homeostatic strengthening of excitatory synapses after chemogenetic silencing of the entorhinal cortex

**Figure 6:**
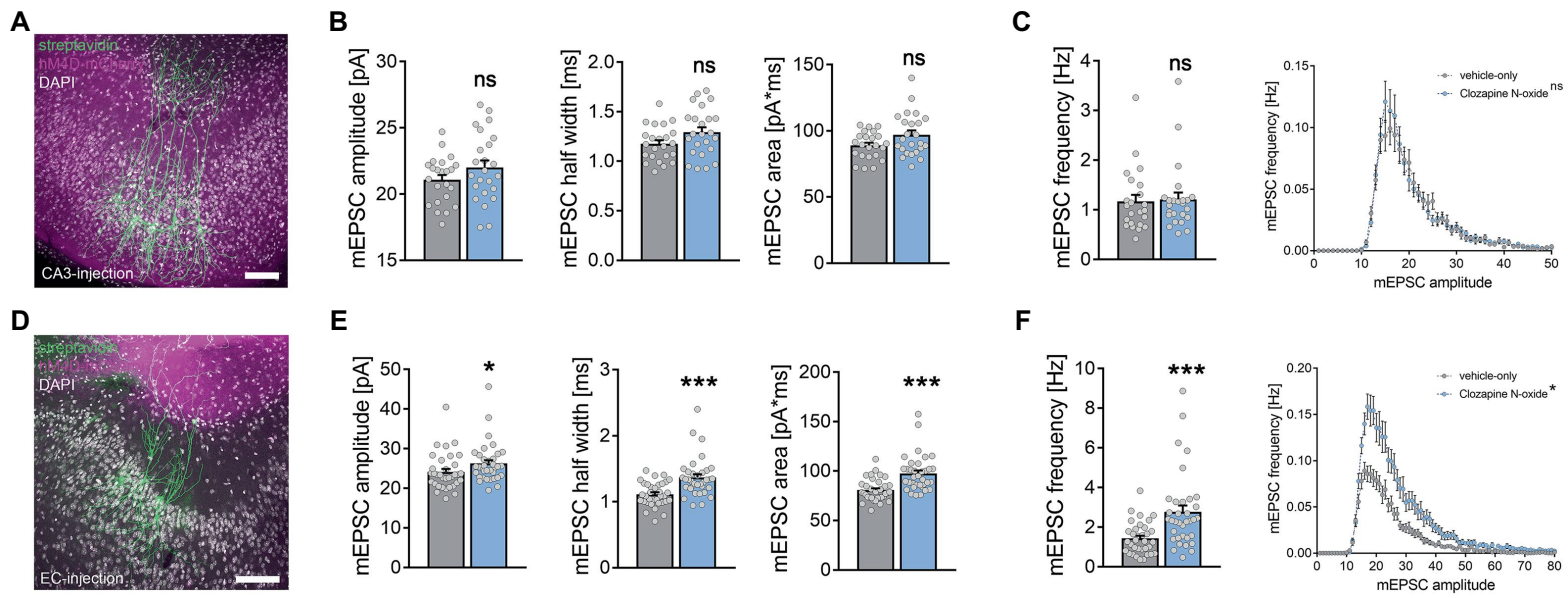
Homeostatic synaptic plasticity in CA1 neurons is specifically induced by chemogenetic silencing of the perforant path. **(A)** Example of recorded and posthoc stained CA1 cells in tissue cultures where viral injection of AAV2-hSyn-hM4D-mCherry was performed in the CA3 region to silence the Schaffer collateral pathway. Scale bar, 100 μm. **(B,C)** Chemogenetic silencing of the Schaffer collateral pathway did not lead to an increase in mEPSC amplitude, area or half width **(B)**. Furthermore, mEPSC frequency and amplitude/frequency analysis reveal no significant differences (**C**, n_control_ = 23 cells, n_CNO_ = 24 cells, Mann–Whitney test for column statistics and RM-two-way ANOVA followed by Sidak’s multiple comparisons test for XY-plot analysis). **(D)** Example of recorded and posthoc stained CA1 cells in tissue cultures where viral injection of AAV2-hSyn-hM4D-mCherry was performed in the entorhinal cortex (EC) to silence the perforant path. Scale bar 100 μm. **(E,F)** Silencing of the perforant path led to a significant increase in mEPSC amplitude, area and half width **(E)**. In line with that, mEPSC frequency was robustly increased and amplitude/frequency analysis revealed significant differences (**F**, n_control_ = 34 cells, n_CNO_ = 35 cells, Mann–Whitney test for column statistics and RM-two-way ANOVA followed by Sidak’s multiple comparisons test for XY-plot analysis). Individual data points are indicated by colored dots. Values represent mean ± s.e.m. (*p < 0.05, ***p < 0.001, ns - non significant difference).

To confirm and extend these results, local viral injections of AAV2-hSyn-hM4D_(Gi)_-mCherry were used to enable the functional silencing of either CA3 or the EC (Figure 6). Three-week old transfected tissue cultures were exposed to clozapine-*N*-oxide (CNO; 100 μM) for 2 days and AMPA-receptor mediated miniature excitatory postsynaptic currents (mEPSCs) were recorded from CA1 pyramidal neurons (Figures 6A,D). In CA3-transfected tissue cultures (Figure 6A), CNO-mediated silencing of Schaffer collateral synapses did not cause any significant changes in excitatory synaptic strength compared to the vehicle-only treated controls (Figures 6B,C). Consistent with the mechanical ECL experiments, CNO silencing of the perforant path in EC-transfected cultures induced significant increases in mean mEPSC amplitude, half width, area, and frequency (Figures 6E,F). We concluded that pathway-specific homeostatic synaptic strengthening was induced after functional and mechanical ECL but not after SCL.

### Denervation-induced RNA oxidation 3 days after SCL and ECL

**Figure 7:**
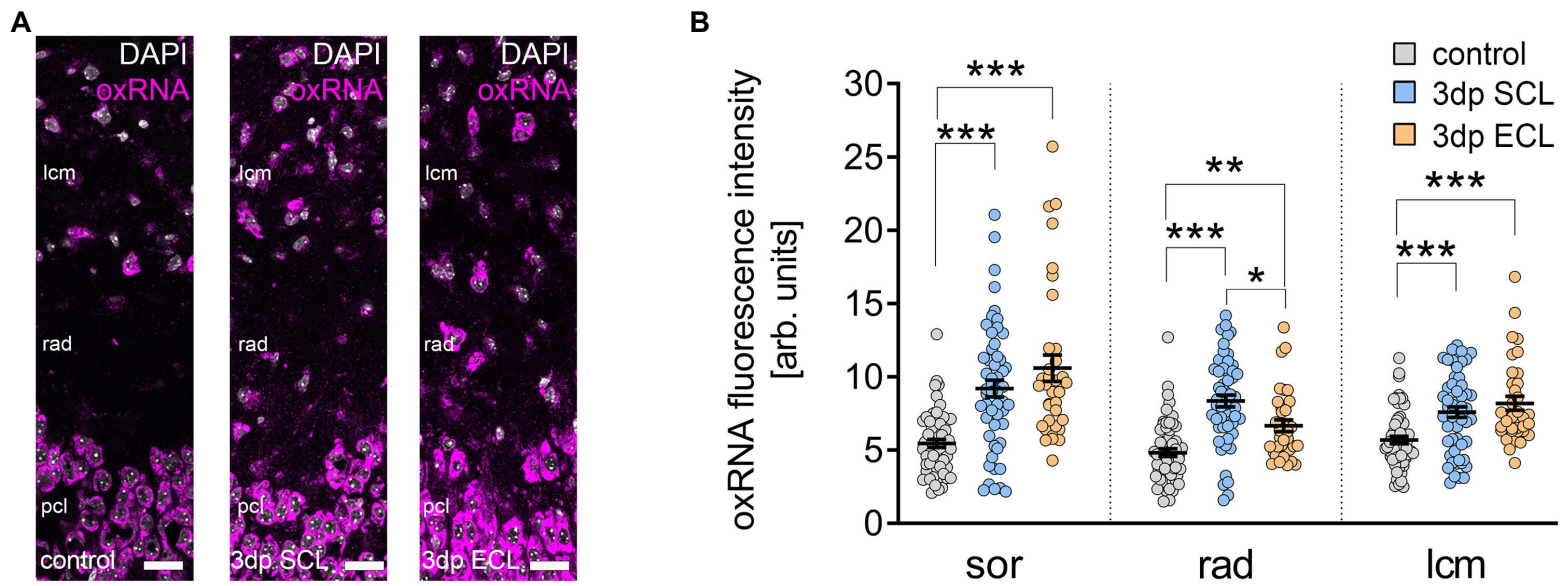
Lesion is accompanied by oxidative RNA modifications in CA1 dendritic layers. **(A)** Representative sample images of the CA1 region of tissue culture controls and 3 days after either SCL or ECL, stained with DAPI nuclear stain and a marker for oxidative RNA modifications. Scale bar, 25 μm. **(B)** The amount of oxidative RNA modifications was significantly increased after both SCL and ECL in all layers of CA1 neurons (n_control_ = 60 visual fields in 12 cultures, n_SCL_ = 55 visual fields in 11 cultures, n_ECL_ = 35 visual fields in 7 cultures, Kruskal-Wallis test followed by Dunn’s posthoc correction). Individual data points are indicated by colored dots. Values represent mean ± s.e.m. (*p < 0.05, **p < 0.01, ***p < 0.001).

The transcriptome analysis identified genes related to oxidative stress 3 days after SCL and ECL, with specific changes related to oxidative modifications observed in the SCL group. Due to the role of local protein translation in synaptic plasticity (Holt et al., 2019), it was assessed whether SCL and ECL were accompanied by RNA oxidation (Figure 7). Immunostaining for oxidated RNA (oxRNA) revealed a significant increase in the fluorescence signal in all layers of the CA1 region 3 days after SCL and ECL compared to non-lesioned controls (Figures 7A,B). Interestingly, the abundance of the oxRNA fluorescence signal in rad was significantly higher in the SCL group compared to the ECL group (Figure 7B). We concluded that SCL and ECL induced global changes in RNA oxidation with pathway-specific differences observed in rad after SCL.

## Discussion

We used mouse entorhino-hippocampal tissue cultures to study the structural, functional, and molecular adaptations of denervated CA1 pyramidal neurons after distinct pathway lesions, i.e., SCL and ECL. Organotypic tissue cultures are characterized by their laminar organization of hippocampal pathways and cortico-hippocampal projections in steady-state conditions (Frotscher and Heimrich, 1993; Heimrich and Frotscher, 1993) that resemble the *in vivo* architecture of synapses at different levels (Maus et al., 2020). Consistent with previous work involving the dentate gyrus (Vlachos et al., 2012, 2013b), (i) microglia accumulation, (ii) degeneration of presynaptic boutons at asymmetric synapses, and (iii) a reduction in dendritic spine numbers were observed in the denervated CA1 layers after SCL and ECL. However, only functional and mechanical ECL did induce homeostatic synaptic strengthening. Both lesions were accompanied by oxidative modification of RNA 3 days after lesion. Our transcriptomic analysis showed complex molecular changes after the lesions, and identified molecular commonalities and differences between SCL and ECL. These results establish a link between transcriptomic changes and the states of synapses after denervation. Specifically, ECL induced enrichment of synaptic gene sets was unique to this form of denervation, indicating functional changes in excitatory neurotransmission that were not observed after SCL. We concluded that denervation-induced homeostatic synaptic plasticity is pathway-specific, and that the lesion-induced activation of microglia following a lesion does not necessarily result in the induction of homeostatic synaptic plasticity in denervated networks.

Microglia interact with neurons through direct contacts (Wake et al., 2009; Masuda et al., 2020; Cserep et al., 2021, 2022) and soluble factors (Stellwagen et al., 2005; Lewitus et al., 2016; Lenz et al., 2020; Eichler et al., 2022). Microglia accumulation in the denervated layers indicated that direct microglia/neuron-interactions (Weinhard et al., 2018; Cserep et al., 2021) and local cytokine release (Lenz et al., 2020) might play a role in denervation-induced plasticity. Previous research demonstrated that activated microglia accumulate in the denervated layers of the dentate gyrus after ECL (c.f. Kleidonas and Vlachos, 2021). Moreover, we previously identified that the pro-inflammatory cytokine tumor necrosis factor alpha (TNFα) plays an important role in the denervation-induced homeostatic synaptic plasticity of dentate granule cells (c.f. Stellwagen et al., 2005; Becker et al., 2013, 2015). These findings support the involvement of microglia in the post-lesional functional and structural reorganization of denervated networks. However, no homeostatic strengthening of excitatory synapses was observed after SCL, despite the accumulation of microglia and reduced spine numbers in the denervated layers. Therefore, a complex role of microglia in denervation-induced homeostatic synaptic plasticity is thought to exist.

Differential activation of microglia in response to SCL and ECL and the concentration-dependent effects of microglial cytokines could play a role in denervation-induced homeostatic synaptic plasticity. Recent research revealed that low concentrations of TNFα promote synaptic plasticity, while high concentrations occlude the ability of neurons to express plasticity (Maggio and Vlachos, 2018; Kleidonas et al., 2022). In line with these results, occlusion of excitatory synaptic plasticity has been reported during inflammation (Strehl et al., 2014; Golia et al., 2019; Lenz et al., 2020; Izumi et al., 2021). Data of the current study demonstrate that microglia accumulate in all dendritic layers except lcm after SCL, while ECL is associated with microglia accumulation specifically in lcm. Whether and how these differences (such as the diffusion of secreted cytokines) account for the pathway-specific induction of homeostatic synaptic plasticity warrants further investigation.

We identified both similarities and differences in the gene expression changes upon SCL and ECL, suggesting that the characteristics of the lesioned pathway play a crucial role in determining postsynaptic gene expression adjustments beyond the common reactive patterns, such as inflammation. This suggests that unique features of neuronal projections influence the ability of postsynaptic neurons to express synaptic plasticity (Blitz et al., 2004; Ho et al., 2011; Lohmann and Kessels, 2014; Grant and Fransen, 2020). However, it is not yet clear which factors are responsible for the diversity of homeostatic adjustments (Lee et al., 2014). While synaptic architecture of specific pathways could account for these observations (O'Rourke et al., 2012; Sancho et al., 2021), recent evidence suggests that glial cells may also be involved in this process, e.g., through differences in their local specializations or by modulating the myelination of axons (Sipe et al., 2016; Jang et al., 2019; Bonetto et al., 2021; De Felice et al., 2022). Additionally, we hypothesize that the predominance of synaptic plasticity in certain pathways may result from the complexity and specialization of neural circuits (Johnson et al., 1996; Dubovyk and Manahan-Vaughan, 2018; Sun et al., 2021). Lesioning distal pathways – here the application of ECL – leads to compensatory changes at proximal synapses, which can be readily detected at neuronal somata, while proximal lesions – here the application of SCL – do not produce compensatory synaptic changes that are detectable at somatic sites. There are various factors that could account for this lack of detectable synaptic adjustments, such as the absence of mRNA transport or excessive oxidative RNA modifications in the denervated layers. Notably, we found a dendritic spine loss in all layers over time irrespective of the lesioned pathway, with more pronounced effects in the denervated layers. In this context, it is interesting to theorize that specific subpopulations of spines across layers are targets for homeostatic synaptic plasticity, e.g., through microglia-synapse interactions (Cramer et al., 2022).

Previous studies demonstrated that RNA modifications can influence RNA stability, transport, and translation efficiency (Boo and Kim, 2020; Hahm et al., 2022). In fact, RNA oxidation is thought to play an important regulatory role in biological systems (Tanaka and Chock, 2021). Oxidative modifications of various RNA forms have been demonstrated to occur under different pathological conditions, such as inflammation or metabolic dysregulation (Fimognari, 2015; Liu et al., 2020; Chen et al., 2022). Furthermore, due to its impact on translation efficiency, where inhibiting actions have been reported (Sochacka et al., 2015; Willi et al., 2018; Thomas et al., 2019), RNA oxidation may interfere with local protein synthesis related to homeostatic synaptic plasticity (Coyle and Puttfarcken, 1993; Nunomura et al., 1999; for references to the role of local protein synthesis in homeostatic synaptic plasticity see Sutton et al., 2004, 2006; Schuman et al., 2006; Sun et al., 2021; Kruse et al., 2022). Therefore, we suggest that inflammation-related oxidative RNA modifications represent a new regulatory mechanism of local homeostatic synaptic plasticity.

Regardless of these considerations, it is possible that SCL-induced local functional changes on distal dendrites (c.f. Aksoy-Aksel and Manahan-Vaughan, 2013; Asgeirsdottir et al., 2020) might not be detectable by somatic patch-clamp recordings, e.g., due to dendritic attenuation (Golding et al., 2005). Nevertheless, our results are consistent with a previous *in vivo* study that showed amplification of CA3-CA1 transmission after ECL and no major functional changes of CA1 activity after SCL (O'Reilly et al., 2014). Likewise, our transcriptomic analysis clearly showed that denervated neurons respond differently after SCL and ECL. We are confident that organotypic entorhino-hippocampal tissue cultures are suitable tools to unravel the cellular and molecular mechanisms that regulate the pathway-specific induction of homeostatic synaptic plasticity operating in complex networks.

## Data availability statement

Source data with statistical evaluations for each figure are provided on the Dryad data repository (https://doi.org/10.5061/dryad.v15dv420w). Raw data (.fastq-files) used for transcriptome analysis are available at the Gene Expression Omnibus; accession number GSE223096. Original data are available from the corresponding authors upon reasonable request.

## Ethics statement

Mice were maintained in a 12 hour light/dark cycle with food and water available *ad libitum*. Every effort was made to minimize distress and pain of animals. All experimental procedures were performed according to the German animal welfare legislation and approved by the animal welfare committee and/or the animal welfare officer at the University of Freiburg, Faculty of Medicine (X-17/07K, X-18/02C, X-21/01B).

## Author contributions

ML: conceptualization, methodology, validation, formal analysis, investigation, writing—original draft preparation, visualization, supervision, project administration, and funding acquisition. AE: validation, formal analysis, investigation, writing—original draft preparation, and visualization. PK: formal analysis, investigation, writing-original draft preparation, and visualization. PS and DK: investigation. CG: investigation and formal analysis. HL: formal analysis, investigation, and visualization. AV: conceptualization, methodology, resources, writing—original draft preparation, supervision, project administration, and funding acquisition. All authors contributed to the article and approved the submitted version.

## Funding

This work was supported by Else Kröner-Fresenius-Stiftung (EKFS_#2019_A94 to ML) and Deutsche Forschungsgemeinschaft (DFG; Project-ID 259373024 B14–CRC/TRR 167 to AV).

## Conflict of interest

The authors declare that the research was conducted in the absence of any commercial or financial relationships that could be construed as a potential conflict of interest.

## Publisher’s note

All claims expressed in this article are solely those of the authors and do not necessarily represent those of their affiliated organizations, or those of the publisher, the editors and the reviewers. Any product that may be evaluated in this article, or claim that may be made by its manufacturer, is not guaranteed or endorsed by the publisher.

## Footnotes

^1^ http://e.informer.com/sys-prog.com

^2^ https://doi.org/10.5061/dryad.v15dv420w

^3^ https://imagej.nih.gov/ij/
