# Supplementary figures and images for: Denervated mouse CA1 pyramidal neurons express homeostatic synaptic plasticity following entorhinal cortex lesion
Source: Front Mol Neurosci. 2023 Apr 12;16:1148219. doi: 10.3389/fnmol.2023.1148219 (PMC10130538; doi:10.3389/fnmol.2023.1148219)

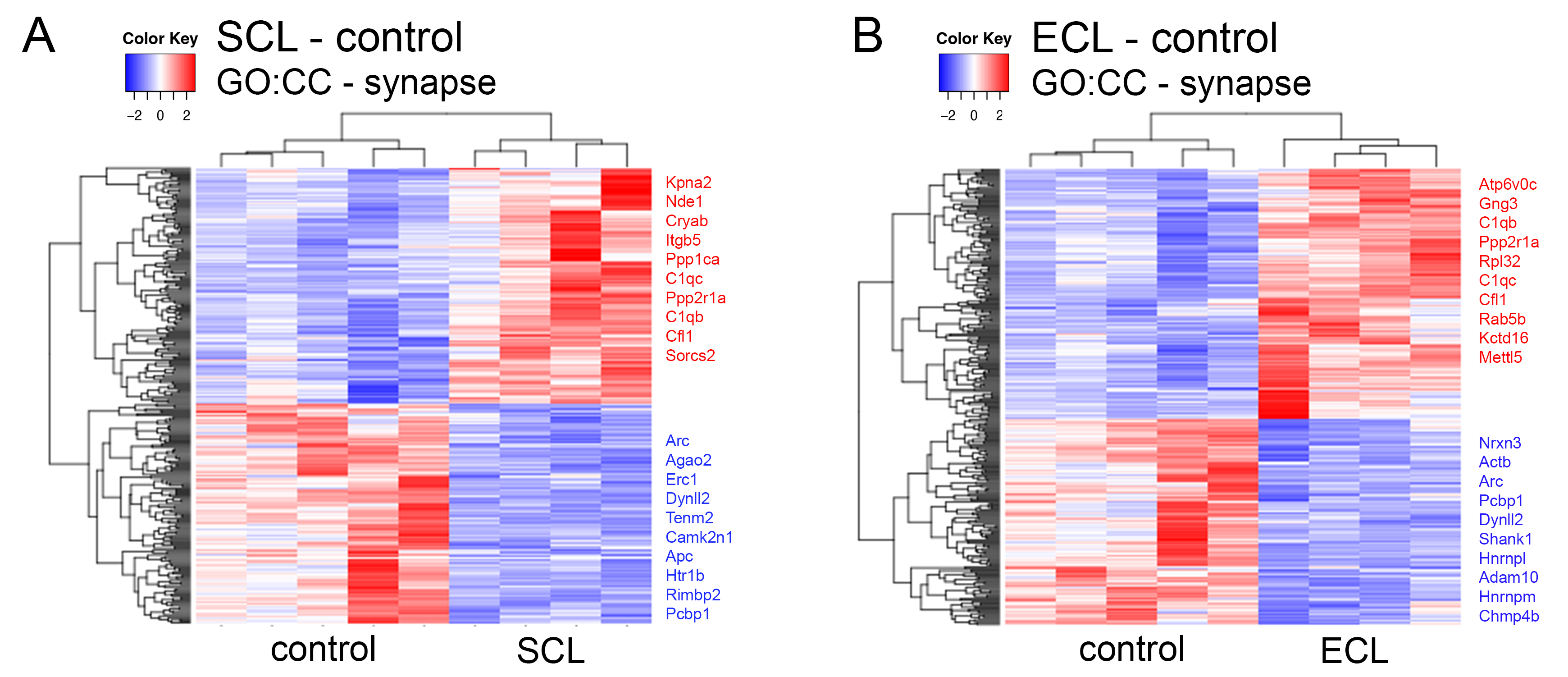

Supplement: Supplementary file 6 [file image_1_v1.tif]
